# Coupling Bioorthogonal Chemistries with Artificial Metabolism: Intracellular Biosynthesis of Azidohomoalanine and Its Incorporation into Recombinant Proteins

**DOI:** 10.3390/molecules19011004

**Published:** 2014-01-15

**Authors:** Ying Ma, Hernán Biava, Roberto Contestabile, Nediljko Budisa, Martino Luigi di Salvo

**Affiliations:** 1 Department of Chemistry, Biocatalysis Group, Technical University Berlin/Berlin Institute of Technology, Müller-Breslau-Str. 10, Berlin 10623, Germany; E-Mails: katrinama2010@gmail.com (Y.M.); biava@chem.tu-berlin.de (H.B.); 2 Dipartimento di Scienze Biochimiche A. “Rossi Fanelli”, Sapienza Università di Roma, Via degli Apuli 9, Roma 00185, Italy; E-Mail: roberto.contestabile@uniroma1.it

**Keywords:** artificial metabolism/metabolic engineering, bioorthogonality, genetic code expansion, posttranslational modifications, l-methionine, l-azidohomoalanine, click chemistry, *O*-acetyl-l-homoserine sulfhydrylase

## Abstract

In this paper, we present a novel, “single experiment” methodology based on genetic engineering of metabolic pathways for direct intracellular production of non-canonical amino acids from simple precursors, coupled with expanded genetic code. In particular, we engineered the intracellular biosynthesis of l-azidohomoalanine from *O*-acetyl-l-homoserine and NaN_3_, and achieved its direct incorporation into recombinant target proteins by AUG codon reassignment in a methionine-auxotroph *E. coli* strain. In our system, the host’s methionine biosynthetic pathway was first diverted towards the production of the desired non-canonical amino acid by exploiting the broad reaction specificity of recombinant pyridoxal phosphate-dependent *O*-acetylhomoserine sulfhydrylase from *Corynebacterium glutamicum*. Then, the expression of the target protein barstar, accompanied with efficient l-azidohomoalanine incorporation in place of l-methionine, was accomplished. This work stands as proof-of-principle and paves the way for additional work towards intracellular production and site-specific incorporation of biotechnologically relevant non-canonical amino acids directly from common fermentable sources.

## 1. Introduction

The main source of chemical diversity in the majority of mature proteins and peptides is due to posttranslational modifications (PTMs), as only a few polypeptide structures have a final covalent structure simply deriving from the translation of their genes [1]. On the other hand, the number of constituent amino acids in ribosome-mediated protein synthesis is restricted to the 20 canonical amino acids. Thus, nature uses PTMs to expand functional diversity, especially in evolutionary advanced organisms such as eukaryotes, mainly metazoans; to this regard, the exploitation of combinatorial PTMs may have been a pre-requisite for the evolution of multicellular complexity [2]. These highly specialized cell forms require a network of membrane structures and compartments to produce additional chemistries essential for sophisticated functions achieved via phosphorylation, glycosylation and other types of post-translational covalent side chain modifications [3]. Mimicking these PTM machineries is not trivial, as many specific recognition features of the protein structure (important for modifications) are easily destroyed during experimental manipulation [4]. In addition, it is notoriously difficult to produce large quantities of homogeneous proteins modified in a specific manner.

Certainly, the most straightforward way to tackle this problem is to insert natural or synthetic non-canonical amino acids (ncAAs) of interest directly during translation, expanding the scope of ribosomal protein synthesis beyond the 20 canonical amino acids–in other words, to re-engineer the genetic code [5]. This process is simpler than PTM itself, as the feature for that particular modification can be based on the unique chemical functionality delivered by the specifically incorporated ncAA, and no longer requires complex signals on the protein scaffold. In an ideal case, many opportunities for chemical coupling come from the integration of specific ncAAs, in particular those with bioorthogonal chemical functionalities such as azides, olefins, carbonyl compounds (ketones and aldehydes), strained- and unstrained-alkynes, halogens, oximes, hydrazones, boronic esters and acids which can subsequently be easily and efficiently coupled with a variety of ligands [6]. Such bioorthogonal conjugations endow proteins with novel and unique functions, leading to improved structural stability (e.g., via cross-coupling or introduction of conformational rigidity [7,8]), specificity, bioavailability and half-life including efficient conjugations with sugars, other peptides, polyethylene glycols, optical markers and so forth [9]. With this method, the biological, chemical and physical properties of the chosen amino acids can be accurately defined by the chemist at the bench. Furthermore, taking advantage of the genetic encoding of these ncAAs, their incorporation into peptides would occur with exquisite fidelity and efficiency. Therefore, instead of exploiting PTMs for expanding the chemical and functional diversity of target proteins, we have now in hands an engineered genetic code to be exploited for the insertion of ncAAs into polypeptides and their subsequent modification.

Among the bioorthogonal chemistries developed for specific chemoselective modifications, the copper(I)-catalyzed Huisgen cycloaddition reaction between azides and alkynes has found widespread application since its mild conditions allow full retention of the protein structure [10,11,12]. This chemistry requires production of recombinant proteins with site- or residue-specific incorporation of alkyne- or azide-containing amino acid analogs by insertion of suitable ncAAs; proteins labeled in this way can be used for reactions with ligands containing complementary azide- or alkyne derivatives. In a typical ncAAs incorporation experiment, the amino acid analog is added to the growth medium and uptaken by the cellular machinery. This is of course perfectly acceptable for small-scale experiments, though it provides no solution for large-scale fermentative production. Thus, the goal is to devise a system where the desired ncAAs are directly produced from medium nutrients, just as all other cell components, to prevent complicated feeding schemes and costly additional substrates. Indeed, many biosynthetic pathways can be identified from different microorganisms which, submitted to genetic engineering, may be exploited to generate novel compounds. For example, the *E. coli* methionine biosynthetic pathway can be diverted at the homoserine level to generate l-azidohomoalanine (Aha), by recombinantly expressing enzymes normally not present in *E. coli*, such as homoserine *O*-acetyltransferase and *O*-acetylhomoserine sulfhydrylase. Significantly, the intermediate of this modified pathway, *O*-acetyl-l-homoserine (Oahs), in not an *E. coli* intermediate and will be totally devoted to the newly engineered metabolism. 

We here demonstrate the feasibility of this approach by describing an auxotrophy-based residue-specific method to introduce Aha into a cysteine-free “pseudo-wild type” barstar (ψB*), a small recombinant protein consisting of 90 amino acids widely used for folding studies. We used two different barstar variants, one containing only one methionine residue (at the *N*-terminus), the other one also containing an internal methionine (Met47). We had previously performed the addition of various labels (sugar, iodine, polyethylene glycols) to the N-terminus of ψB* containing either azide or alkyne moieties, delivering novel functional features while retaining original barstar structure and function [8,13]. In this work, we repeat the *N*-terminal addition of a dansyl fluorescent label to the recombinantly expressed Aha-modified ψB*.

## 2. Results and Discussion

### 2.1. Semisynthetic Production of l-Azidohomoalanine

**Figure 1:**
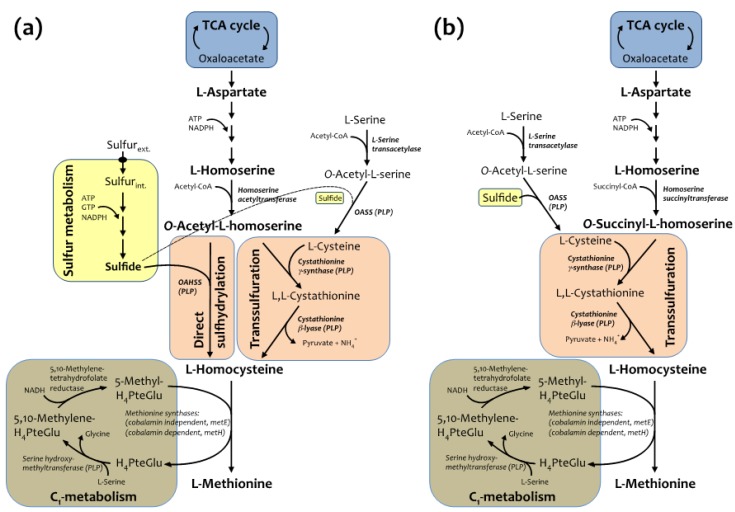
(**a**) Methionine biosynthetic pathway in *C. glutamicum* and (**b**) *E. coli*.

It has been previously shown that *O*-acetylhomoserine sulfhydrylase (OAHSS), the sulfide-utilizing enzyme involved in the direct sulfhydrylation pathway of l-methionine (Met) biosynthesis in various bacteria and yeasts (Figure 1a) [14,15,16], displays a somehow relaxed substrate specificity [15,17,18]. Similarly, its more characterized cognate enzyme, *O*-acetylserine sulfhydrylase (OASS), involved in l-cysteine biosynthesis, also accepts several types of thiols and selenols as substrate nucleophiles, as well as azide, cyanide and aromatic five-membered heterocycles containing at least two neighboring nitrogen atoms [19]. Interestingly, recent biochemical studies have suggested that the likely sulfur source utilized by OAHSS in the methionine biosynthetic pathway may differ from organism to organism. In *W. succinogenes*, for example, the sulfur source is a protein thiocarboxylate, so that the sulfur atom of methionine is incorporated by OAHSS using a sulfur-carrier protein rather than sulfide, as it is the case for other OAHSSs [20]. Methionine is an essential amino acid serving diverse roles: besides being a proteinogenic amino acid, methionine is an intermediate in the biosynthesis of cysteine, carnitine, taurine, lecithin, phosphatidylcholine, and other phospholipids, and its derivative *S*-adenosyl methionine is a universal methyl donor [21]. l-methionine biosynthesis in *C. glutamicum* is carried out by two parallel pathways: transsulfuration and direct sulfhydrylation (Figure 1a, [15]).

Both pathways utilize l-homoserine as precursor (originating from l-aspartate) leading to production of *O*-acetyl-l-homoserine (Oahs), the diverging point of the two branches, which will converge again at l-homocysteine level. The last step of methionine biosynthesis is the folate-dependent methylation of homocysteine, carried out by two distinct catalytic activities (one of which is dependent on vitamin B_12_). Methionine metabolism draws precursors from TCA cycle, C_1_ and sulfur metabolisms and requires energy, reducing power and cofactors such as pyridoxal 5'-phosphate (PLP), flavin, coenzyme A (CoA) and folates. The biosynthetic pathway of methionine in *E. coli* is similar to the one described for *C. glutamicum*, but differs in few important steps (Figure 1b). First of all, direct sulfhydrylation does not operate in *E. coli*, and sulfur is incorporated mainly into l-cysteine, through the reaction catalyzed by OASS. Secondly, Oahs is not an *E. coli* metabolite, for this organism is able to only use *O*-succinyl-l-homoserine as substrate for cystathionine γ-synthase in the transsulfuration pathway. Following this scheme, recombinantly expressed *cg*OAHSS may be exploited in *E. coli* for intracellular production of Met analogs, starting from Oahs and selected nucleophiles.

**Scheme 1:**
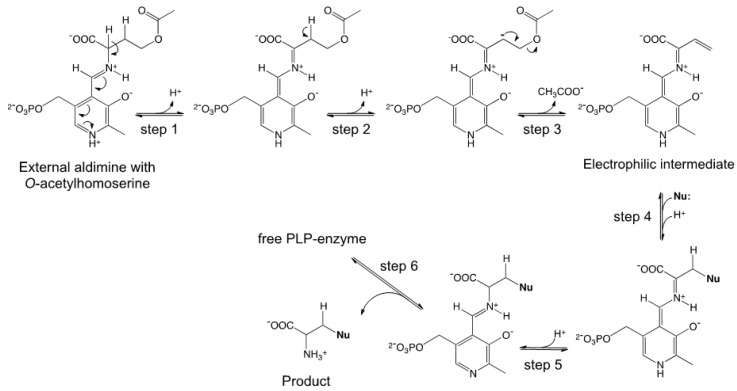
Proposed reaction mechanism for *O*-acetylhomoserine sulfhydrylase (OAHSS).

OAHSS is a PLP-dependent enzyme belonging to the fold-type II subclass, a family of strictly related enzymes sharing the same protein fold and catalyzing diverse side-chain substitutions on amino acid substrates; well-known representatives of this group of enzymes are, among others, the β-subunit of tryptophan synthase and OASS [22]. These enzymes, besides being crucial in human health and envisaged targets for therapeutic agents, have great biotechnological potential and can been employed for amino acid analogs synthesis [19,22,23]. The reaction mechanism of OAHSS, as hypothesized by Tran *et al*. [24], is shown in Scheme 1.

The initial steps, up to formation of the external aldimine (in which the substrate Oahs is bound to the cofactor at the enzyme active site), are common to all PLP-dependent enzyme-catalyzed reactions [25]. The Oahs external aldimine then undergoes a double deprotonation reaction (steps 1–2), followed by release (γ-elimination) of acetate (step 3), to form a key electrophilic intermediate (imino-3-butenoate). The reaction then proceeds through the addition of an upcoming nucleophile (e.g., a sulfide ion, S^2−^) to this intermediate (step 4): the product is subsequently released from the cofactor and from the enzyme (steps 5–6). The overall reaction can be seen as a γ-substitution on Oahs.

**Figure 2:**
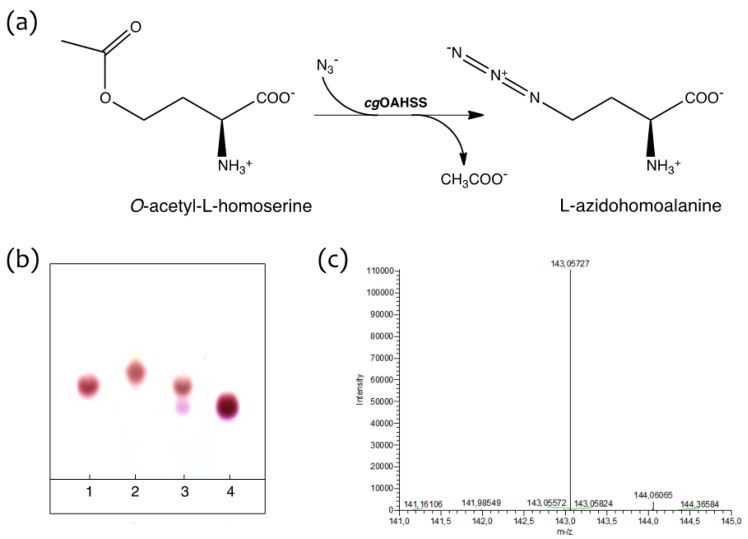
Enzymatic production of l-azidohomoalanine (Aha). (**a**) Reaction scheme; (**b**) Ninhydrin stained thin-layer chromatography showing the OAHSS-catalyzed synthesis of Aha. Lane 1: Oahs + NaN_3_ in absence of *cg*OAHSS; lane 2: Oahs + NaN_3_ in presence of the enzyme; lane 3: Oahs only, in presence of the enzyme; lane 4: l-homoserine; (**c**) ESI-HRMS analysis of enzyme-produced Aha (negative mode).

Making advantage of the above-mentioned relaxed substrate and reaction specificity of OAHSS (indeed a quite distinctive feature of PLP-dependent enzymes [26,27], the ability of *cg*OAHSS to use azide as reacting nucleophile and catalyze the formation of l-azidohomoalanine from *O*-acetyl-l-homoserine (Figure 2a) was tested *in vitro*. *O*-acetyl-l-homoserine was synthesized as described in the Experimental section. *C. glutamicum* OAHSS was overexpressed in rich growing media in *E. coli* DH5α strain. The expression of *cg*OAHSS gene, cloned into the pBU26'1GK vector (to generate the construct pBU26'1GK-*metY*-HTC) was directed by a constitutive mutant *glnS*' promoter [28]. OAHSS was either purified to homogeneity by means of His-tag affinity chromatography or directly used as clarified cell extract. Small-scale reactions were monitored by thin-layer chromatography, following the disappearance of the amino acid substrate and the appearance of a higher R_f_ spot (Figure 2b). Oahs (10 mM) and NaN_3_ (10 mM) in 20 mM sodium HEPES buffer pH 7.2 (final volume 25 µL) were maintained at 37 °C in the absence (lane 1) or presence (lane 2) of 5 µM purified *cg*OAHSS. A control reaction was carried out in which the enzyme was incubated with Oahs only (lane 3). After one hour, 1.5 µL samples were analyzed. It is clear that, whether present as purified enzyme or as clarified cell extract, OAHSS efficiently catalyzed the complete conversion of *O*-acetyl-l-homoserine to l-azidohomoalanine. It should be also noticed that the enzyme, in the absence of NaN_3_, slowly catalyzes the slow hydrolysis of Oahs into l-homoserine, as showed by the presence of a minor spot in lane 3 with an R_f_ corresponding to that of a standard sample of this amino acid (lane 4).

A larger scale reaction mixture was EtOH-precipitated and the supernatant was directly analyzed by HPLC-ESI-HRMS, giving the expected molecular weight of azidohomoalanine (Figure 2c). The reaction product was further characterized by ^1^H-NMR, ^13^C-NMR, IR and experimental data were in agreement with previously reported chemical synthesis [29,30] (see Supplementary Information, Figures S3 and S4).

### 2.2. *In Vivo* Incorporation of l-Azidohomoalanine into Barstar

The recombinant activity of *cg*OAHSS carried by the pBU26'1GK-*metY*-HTC vector can be exploited for the production of Aha and its incorporation into upgrowing proteins directly inside *E. coli* cells. To this aim, we used *E. coli* B834 strain, a protease-deficient, methionine auxotroph host normally used for high specific-activity labeling of target proteins with ^35^S-Met and selenomethionine for crystallography purposes. In our case, it will allow specific incorporation of Aha, in place of Met, into the host produced proteins. It was previously demonstrated that various Met analogs, including Aha, could be introduced into target proteins by supplementation-based incorporation through AUG codon reassignment [31,32,33]. In the present study, we used an engineered triple mutant, cysteine-free “pseudo wild-type” barstar form (Pro28Ala/Cys41Ala/Cys83Ala; abbr. ψB*) as target protein [34]. Barstar is the intracellular inhibitor of barnase, an extracellular RNAse from *B. amyloliquefaciens*; it is a small single domain protein consistent of 90 amino acids, widely used for folding studies. Its only Met residue, present at the *N*-terminal position, does not get cleaved upon expression in *E. coli* cells [33]. Hereinafter, this barstar form (containing only the *N*-terminal methionine) will be referred to as 1Met-ψB* to be distinguished from the ψB* E47M mutant form, containing two Met residues, which was also used as target protein for further testing our incorporation system (this latter form is referred to as 2Met-ψB*). The barstar forms in which Aha is incorporated instead of methionine will be designed as 1Aha-ψB* and 2Aha-ψB*, respectively.

**Figure 3:**
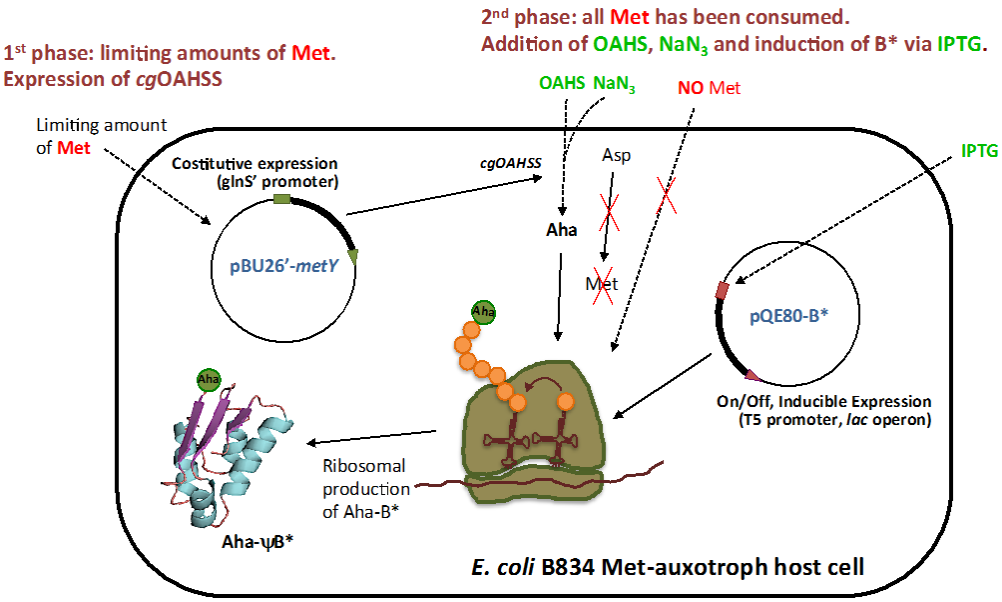
General method for intracellular l-azidohomoalanine production and incorporation into recombinant proteins. The procedure is described in details in the text.

The general method for intracellular production and incorporation of Aha into target proteins is shown in Figure 3. In a first phase, an overnight culture of Met-auxotroph *E. coli* strain is grown in minimal medium supplemented with sizeable amounts of 19 amino acids (0.5 mM) but limiting amount of Met (0.045 mM), just enough to reach mid-log phase growth (OD_600_ of 0.6–0.8), at which point Met will result depleted from the medium. During this period, an adequate amount of viable cells is generated and recombinant *cg*OAHSS is expressed. In the second phase, the substrates of OAHSS are added (in our case Oahs and NaN_3,_ both at final concentration of 1 mM) and the growth is continued for another hour in order to allow for enzymatic, intracellular production of Aha. The last step consists in the addition of IPTG to induce ψB* expression. The growth is then carried over for 4–5 more hours to produce and accumulate recombinant Aha-ψB*. A positive control in which Met (0.3 mM) is added instead of Oahs and NaN_3_ was also performed to check for natural barstar expression (Met-ψB*). It is important to note that, although NaN_3_ is well known to acts as a bacteriostatic by inhibiting cytochrome oxidase in gram-negative bacteria, under our experimental conditions *E. coli* was capable to sustain enough cell growth and viability for protein expression.

**Figure 4:**
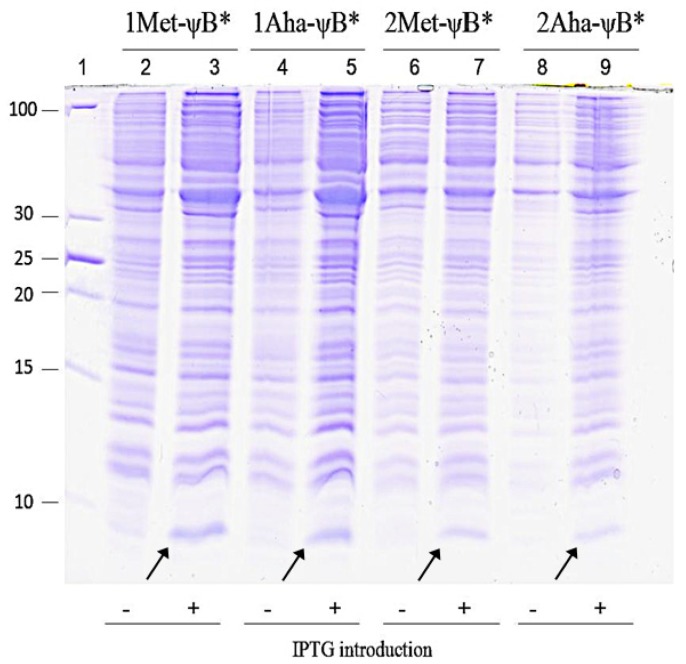
Coomassie-stained 20% SDS-PAGE showing Met-ψB* and Aha-ψB* expression. Aha-ψB* refers to ψB* expressed in presence of Oahs and NaN_3_, whereas Met-ψB* refers to the positive control, in which Met was added to the system. Lane 1: PageRuler Unstained Low Range Protein Ladder (Thermo Fisher Scientific); molecular weights are indicated in the figure. Lane 2, 4, 6 and 8: cell extract before induction; lane 3, 5, 7 and 9: cell extract after induction for Met-ψB* and Aha-ψB*, as indicated on the top of the figure. The arrows indicate the 10 kDa protein band attributable to IPTG-induced barstar expression.

The most direct evidence that every step of the manufacturing system described in Figure 3 is suitably working, (production of active *cg*OAHSS, efficient rate of OAHS and NaN_3_ uptake, consistent incorporation of Aha and expression of properly folded target protein), is to verify the expression of ψB* and characterize its physical and chemical properties. Figure 4 shows the expression of Met-ψB* and Aha-ψB* upon IPTG induction. The evident 10 kDa Coomassie-stained band in lanes 3, 5, 7 and 9 clearly indicates a good production of recombinant ψB*. All forms (1 and 2Met-ψB*, 1 and 2Aha-ψB*) were expressed to a similar level and were promptly purified by means of anion-exchange chromatography for further analyses. From each 0.5 L of bacterial culture we were able to extract about 2.8 mg of azido-labeled barstar, comparable to previously reported data in which Aha was added directly into the growing medium [33]. The yield of purified Aha-ψB* was about 56% of that for Met-ψB*.

**Figure 5:**
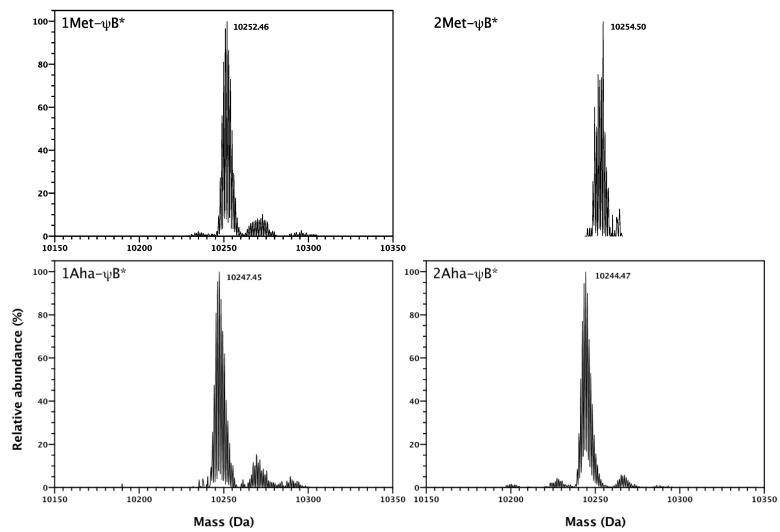
Deconvoluted ESI-MS spectra of 1Met-ψB*, 1Aha-ψB*, 2Met-ψB* and 2Aha-ψB* forms. The predicted molecular weights of are: 10,252.92, 10,254.99, 10,247.84 Da, and 10,244.83 Da, respectively. The measured molecular weights are shown in the figure.

The incorporation of Aha in ψB*, at both *N*-terminal and position 47 was confirmed by electrospray ionization mass-spectrometric analysis (ESI-MS) (Figure 5). For each Met→Aha substitution, the molecular weight of the purified protein should decrease of about 5 daltons (M.W. Met = 149, M.W. Aha = 144). The mass spectra of natural Met-ψB* and substituted Aha-ψB* forms are in agreement with the predicted molecular weight (see legend of Figure 5) and revealed a high level of replacement. The minor peaks visible in Figure 5 are most probably unspecifically bound cation adducts arising from sodium present in the buffer (*ca.* +22 Da). Significantly, the system showed high incorporation efficiency also for barstar containing more than one methionine residue, demonstrating that the ribosome translation system is able to incorporate Aha at both initial and internal AUG codons. These data also confirm that the presence of bulky amino acids in position two and three of barstar sequence protects the *N*-terminal Aha from being excised by the methionylaminopeptidase bacterial system [33]. The presence of *N*-formylmethionine at the *N*-terminal position can be excluded, since no signal corresponding to the predicted molecular mass of the formylated species (10,275–10,282 Da) can be detected.

**Figure 6:**
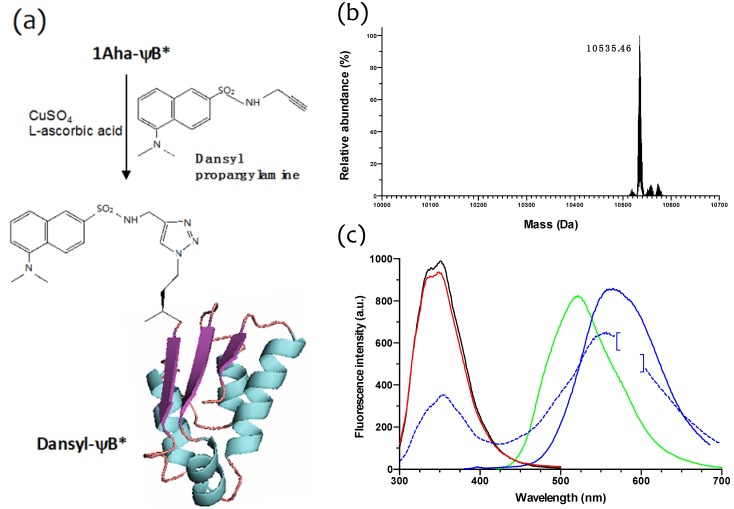
Click chemistry on 1Aha-ψB* and fluorescence spectra of different ψB* variants. (**a**) Scheme of copper(I)-catalyzed Huisgen [3+2] cycloaddition of dansyl proparglylamine to 1Aha-ψB* and formation of *N*-terminal labeled dansyl-ψB*; (**b**) Deconvoluted ESI-MS spectrum of dansyl-ψB*. The measured molecular weight is shown in the figure. (**c**) Fluorescence emission spectra of 1Met-ψB* (black line), 1Aha-ψB* (red line), dansyl-ψB* (blue solid line) upon excitation at 290 nm, and of dansyl-ψB* (blue dotted line) and free dansyl propargylamine (green line) upon excitation at 350 nm. The data point between 575 and 600 nm relative to the blue solid line spectrum were taken out (brackets) due to strong second order diffraction grating effect.

In order to check the correct fold of recombinantly expressed barstar, the effects of Aha→Met substitutions on its structural properties were analyzed by means of spectrofluorimetry, circular dichroism and thermal stability. CD spectra and thermal denaturation curves relative to Aha-ψB* forms were very similar to the methionine-containing proteins and are comparable to previously reported data [33], indicating that incorporation of Aha did not alter the secondary structure and the correct fold of barstar (data not shown). Fluorescence spectra are shown in Figure 6 and discussed in the following paragraph.

### 2.3. Click Chemistry Derivatization of 1Aha-ψB*

In order to check the chemical reactivity of the *N*-terminal Aha residue of 1Aha-ψB* and site-specifically introduce a fluorescent probe, a sample of purified protein was subjected to copper(I)-catalyzed Huisgen [3+2] cycloaddition with dansyl propargylamine, as shown in Figure 6a. The reacted sample (designated as dansyl-ψB*) was then extensively dialyzed and analyzed by ESI-MS, providing a well-defined peak of 10,535.46 Da (Figure 6b), consistent with the predicted molecular weight. The fluorescence properties of 1Met-, 1Aha- and dansyl-ψB* forms were then characterized (Figure 6c). When excited at 290 nm, 1Met- and 1Aha-ψB* both showed a fluorescence emission band with a maximum at 350 nm, due to the intrinsic fluorescence of the three tryptophan residues present in barstar (black and red lines, respectively). The shape and amplitude of these bands were comparable, demonstrating that the overall tertiary structure had been conserved between the wild type (1Met-ψB*) and the Aha-substituted form. On the other hand, when excited at 290 nm, the dansyl-ψB* sample (blue solid line) showed a reduced 350 nm band and an additional pronounced emission band at 560 nm, characteristic of its dansyl fluorophore when this is excited at 350 nm (blue dotted line; the emission band of free dansyl propargylamine, excited at 350 nm, is shown as a green line and shows a maximum at around 525 nm). Therefore, the occurrence of the 560 nm emission band when the dansyl-ψB* protein is excited at 290 nm has to be attributed to fluorescence resonance energy transfer (FRET) between a nearby tryptophan residue and the *N*-terminal dansyl moiety. These results clearly show that the dansyl fluorophore has been specifically added to 1Aha-ψB*.

The 1Aha- and 1Met-ψB* samples do not show any 560 nm emission peak neither when excited at 290 nm nor at 350 nm (data not shown in the figure). Furthermore, the emission spectra or the 2Aha- and 2Met-ψB* variants are superimposable to the 1Aha- and 1Met-ψB* forms (not shown). All together, these data confirm that the Aha derivatives of ψB* retain the barstar native structure.

## 3. Experimental

### 3.1. Chemical Synthesis and Analysis

Reagents and chemicals were purchased from Sigma-Aldrich (St. Louis, MO, USA) and used without further purification. Solvents were purified by simple distillation. Nuclear magnetic resonance spectra were recorded at room temperature on a Bruker Avance 400 MHz instrument using standard deuterated solvents (Billerica, MA, USA). IR spectra were recorded on a Jasco FT/IR-4100 (Jasco Inc., Easton, MD, USA). Samples were run as KBr pellets. HPLC-ESI-HRMS was performed on a LTQ Orbitrap XL using a Syncronis C18 reversed-phase analytical column (Thermo Fisher Scientific, Waltham, MA, USA), length 50 mm, ID 3 mm, 5 µm. A 5% to 100% solvent 2 gradient in 12 min was employed for elution. Solvent 1: H_2_O, 0.025% HCO_2_H. Solvent 2: MeOH, 0.025% HCO_2_H. Flow rate: 1.3 mL min^−1^. Analytical thin layer chromatography (TLC) was performed on Silica Gel 60 F_254_ aluminum sheets (Merck KGaA, Darmstadt, Germany) and developed by a n-butanol/acetic acid/water 3:1:1 (by volume) solution. Compound spots were visualized by quenching of fluorescence and/or by charring after treatment with ethanolic ninhydrin (3% w/v).

### 3.2. Synthesis of O-Acetyl-l-homoserine

**Scheme 2:**
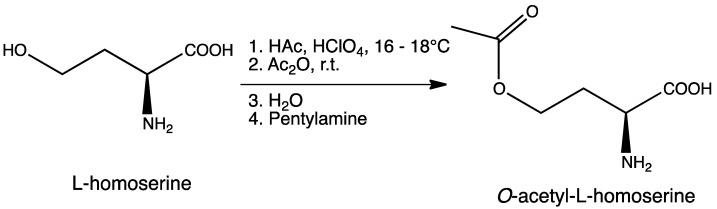
Synthesis of *O*-acetyl-l-homoserine (Oahs).

This compound was synthesized according to a published procedure with some modifications ([35], Scheme 2).

To a round bottom flask containing a mixture of glacial acetic acid (99.9%, 20.0 mL, 345.9 mmol) and perchloric acid (60%, 1.7 mL, 43.3 mmol) cooled at 16–18 °C, was carefully added acetic anhydride (99%, 8.1 mL, 84.8 mmol) under stirring, followed by the addition of l-homoserine (1.0 g, 8.4 mmol) previously dissolved in acetic acid (10.0 mL). The reaction was allowed to reach room temperature and stirred for 90 min. Unreacted acetic anhydride was then quenched by addition of water (0.22 mL) and the mixture was neutralized with pentylamine (2.0 mL, 17.2 mmol). After adding diethyl ether (300 mL), the crude was kept overnight at 4 °C and then five more hours at −20 °C. A precipitate of homoserine lactone was formed and removed by filtration. Filtrate was diluted with water (15 mL), filtered again, and finally diluted in EtOH. After standing overnight at 4 °C, a second precipitate was obtained. This product was isolated by filtration and recrystallized twice from EtOH to give 0.82 g (5.1 mmol, 61% yield) of *O*-acetyl-l-homoserine as a white solid. ^1^H-NMR (400 MHz, D_2_O) *δ* 4.23 (t, *J* = 6.0 Hz, 2H), 3.84 (dd, *J*_1_ = 6.8 Hz, *J*_2_ = 5.2 Hz, 1H), 2.32–2.15 (m, 2H), 2.09 (s, 3H); ^13^C-NMR (100 MHz, D_2_O) *δ* 174.4, 174.2, 61.8, 53.0, 29.5, 20.6; IR (KBr), ν_max_ 3434, 3168, 2969, 2630, 1743, 1616, 1588, 1564, 1595, 1414, 1389, 1249, 1052 cm^–1^; HRMS (ESI-MS) *m/z*: (M + H)^+^ calculated for C_6_H_12_NO_4_ = 162.0766; found 162.0758 (see Supplementary Information, Figures S1,S2 and S4).

### 3.3. Synthesis of l-Azidohomoalanine

The enzymatic synthesis of Aha was carried out in 20 mM sodium HEPES (4-(2-hydroxyethyl)-1-piperazineethanesulfonic acid), pH 7.2, 37 °C as described in the text. Quantitative conversion of *O*-acetyl-l-homoserine into l-azidohomoalanine was achieved as judged by TLC analysis. To get rid of protein fraction, the reaction mixture was EtOH-precipitated, then freeze-dried to completeness. The compound was obtained as a colorless powder. ^1^H-NMR (400 MHz, D_2_O) *δ*: 3.8 (m, 1H) 3.55 (m, 2H) 2.11 (m, 2H); ^13^C-NMR (100 MHz, D_2_O) *δ* 173.9, 52.9, 47.5, 29.5; IR (KBr), ν_max_ 3429, 2953, 2621, 2110, 1614, 1587, 1509, 1412, 1356, 1251, 1157, 1091 cm^–1^; HRMS (ESI-MS) *m/z*: (M − H)^−^ calculated for C_4_H_7_N_4_O_2_ = 143.0574; found: 143.0573 (see Figure 2 and Supplementary Information, Figures S3 and S4).

### 3.4. Molecular Biology and Plasmid Construction

Oligonucleotides and DNA sequencing were from Eurofins MWG Operon (Ebersberg, Germany). Ni-NTA Agarose for purification of His-tagged proteins was from Qiagen Inc. (Valencia, CA, USA). Ingredients for bacterial growth and all other chemicals were from Sigma-Aldrich (St. Louis, MO, USA) and Carlo Erba Reagents (Milano, Italy). *E. coli* B834 strain (F^−^*ompT hsdS_D_*(*rd−md−*) *gal dcm metE*) was from Novagen Merck Chemicals (Darmstadt, Germany).

The DNA fragment containing *metY* sequence encoding *Corynebacterium glutamicum O*-acetylhomoserine sulfhydrylase (GenBank: FJ483537.1) linked to a C-terminal poly-histidine tag (*metY*-HTC) was obtained from Jörn Kalinowski and Alfred Pühler, Center for Biotechnology, Bielefeld University, Germany. The coding sequence of *metY*-HTC was amplified by PCR using the following oligonucleotides: forward primer: 5'-CGCCGCTAGCCCAAAGTACGACAATTCCA-3'; reverse primer: 5'-AAAAGGCGCCCTAGATTGCAGCAAAGCCGCC-3'. The resulting 1300 bp DNA fragment was inserted into vector pBU26'1GK (a cargo site modified derivative of pSEVA26'1 from the SEVA collection [36]), between *Nhe*I and *Kas*I restriction sites (underlined in the above primer sequences) to put the gene under control of the strong constitutive *glnS*' promoter [28]. The pBU26'1GK vector was kindly obtained from Dr. Michael Hösl and M.Sc. Jan Völler (Department of Chemistry, Biocatalysis Group, Technical University Berlin/Berlin Institute of Technology, Germany). The nucleotide sequence of the insert was determined to confirm that no mismatching had occurred during the PCR amplification.

The coding sequence of triple mutant, cysteine-free “pseudo wild-type” barstar form (ψB*) [34], containing only one Met residue at the *N*-terminal position (1Met-(ψB) was cloned into pQE80L vector between the *EcoR*I and *Hind*III resctriction sites. In this construct, expression of target protein is driven by coliphage T5 promoter, recognized by *E. coli* RNA polymerases, and is under control of *lac* operon, inducible with the addition of isopropyl-β-d-1-thiogalattopyranoside (IPTG). A similar construct was prepared, in which the mutant form E47M of ψB*, containing two methionine residues (2Met-ψB), was likewise cloned into pQE80L vector.

### 3.5. Purification of C. glutamicum O-Acetylhomoserine Sulfhydrylase

A 1.5 L culture of *E. coli* DH5α cells transformed with plasmid pBU26'1GK-*metY-*HTC was grown overnight in Luria-Bertani broth containing kanamycin (40 g mL^−1^) at 37 °C under constant shaking, in Erlenmeyer flasks. Bacteria were harvested after 18 h and suspended in 50 mL of 50 mM potassium phosphate buffer at pH 7.2 containing 150 mM NaCl, 1 mM EDTA (ethylenediaminetetraacetic acid), 2 mM 2-mercaptoethanol and SIGMAFAST^™^ protease inhibitor (half cocktail tablet). Cell lysis was carried out by the addition of 10 mg lysozyme, incubated for 30 min at room temperature, followed by freezing, thawing and sonication on ice (2-min in short 5-s pulses with 20-s intervals). Lysate was centrifuged at 18,000 g for 30 min and the pellet was discarded. At this point, an aliquot of the supernatant was withdrawn and directly used for enzymatic reactions (clarified cell extract). The rest of supernatant was added to a Ni-NTA agarose column (1 cm × 3 cm), previously equilibrated with 50 mM potassium phosphate buffer at pH 7.5, containing 150 mM NaCl. The column was washed with 100 mL of the same buffer and eluted with a linear 0 to 300 mM imidazole gradient (the mixing chamber contained 100 mL of the equilibration buffer plus 100 mL of the same buffer including 300 mM imidazole; the buffer containing imidazole, was adjusted to pH 7.5 with HCl). Collected fractions were analyzed by SDS-PAGE and those containing the target protein (M.W. 46 kDa) were pooled and dialyzed overnight against 2 L of 20 mM potassium phosphate buffer at pH 7.2, containing 0.2 mM dithiothreitol (DTT). The enzyme was stored at −20 °C. Enzyme subunit concentration was calculated on the basis of PLP content [37] and using a theoretical extinction coefficient at 280 nm of 47,900 M^−1^ cm^−1^ (calculated with the Expasy ProtParam tool).

### 3.6. Expression and Purification of the Met- and Aha- Variants of Barstar

Plasmids pQE80L containing the ψ coding sequence were transformed into the Met-auxotrophic *E. coli* B834 strain, which already contained the pBU26'1GK-*metY*-HTC vector. For the expression of Met→Aha-substituted ψ (Aha-ψB*), as well as the natural Met ψB variants (Met-ψB*), we followed a previously described protocol [33]. Briefly, cells were grown to mid-log phase (OD_600_ at about 0.6–0.8) in 0.5 L of synthetic new minimal medium (NMM [38]) supplemented with the appropriate antibiotics (100 µg mL^−1^ ampicillin for pQE80L and 40 µg mL^−1^ kanamycin for pBU26'1GK selection, respectively), and containing non-limiting concentration of the 19 amino acids (0.5 mM) plus limiting amounts (0.045 mM) of Met. Growths went typically overnight at 30 °C under constant shaking. The Met-starved cultures were then supplemented with either Met (0.3 mM), or Aha and NaN_3_ (both at 1 mM) and were let grow for 1 more hour, after which, the expression of barstar was induced by adding IPTG to a final concentration of 0.5 mM. The overexpression of Aha-ψB* and Met-ψB* were verified by 20% SDS-PAGE and the target proteins were then purified by ion-exchange chromatography. Cells were first harvested and lysed in lysis buffer (50 mM potassium phosphate, 300 mM NaCl, pH 8.0). After addition of lysozyme, DNAse and RNAse, the suspension was sonicated and spun down (18,000 *g*, 4 °C, 45 min). The supernatant was extensively dialyzed against 50 mM Tris-HCl (pH 8.0), then loaded onto HiTrap Q-sepharose (GE Healthcare Bio-Sciences AB, Uppsala, Sweden), washed (50 mM Tris-HCl, pH 8.0) and eluted (gradient from 0 to 1 M NaCl in the previous buffer). Elution fractions were analyzed by SDS-PAGE, and those enriched in the desired protein were pooled and subsequently analyzed.

### 3.7. Analysis of Barstar Variants

Protein samples containing purified Aha-ψB* and Met-ψB*s were examined by high-resolution mass spectrometry performed on a Exactive Orbitrap Mass Spectrometer (Thermo Fisher Scientific). Purified proteins were injected directly in a carrier flow of 30% A (0.05% TFA in water) and 70% B (0.05% TFA in acetonitrile) at a rate of 250 µL min^−1^.

Fluorescence spectra of protein samples were measured on a luminescence spectrophotometer LS 50 B (PerkinElmer Life Sciences, Boston, MA, USA) using excitation and emission slits of 5 nm. The concentration of the samples was 0.8 µM. The proteins were excited at 290 nm and 350 nm and the fluorescence was measured at 20 °C in a range of 300 to 700 nm. 

Circular dichroism spectra of ψB* variants were recorded on a JASCO J-715 Spectropolarimeter equipped with a Peltier type temperature control attachment (Jasco Inc., Easton, MD, USA) at a protein concentration of 15 µM, in a 110 QS Hellma quartz cuvette with an optical path length of 0.1 cm. Melting curves of ψB* variants were measured at a concentration of 0.2 mg mL^−1^ by monitoring the changes in residual ellipticity at 220 nm. The protein solutions were heated from 4 to 95 °C with a rate of 30 °C h^−1^. At 95 °C and after cooling back to 4 °C, CD spectra were measured again to analyze denaturation and renaturation.

### 3.8. Copper(I)-catalyzed Huisgen [3+2] Cycloaddition

Click chemistry to generate *N*-terminal labeled ψB* was performed as follow: the reaction mixture consisted of 1Aha-ψB* (100 µL, 8 mg mL^−1^ in 100 mM phosphate buffer, pH 8.0, final concentration 1.6 mg mL^−1^), dansyl propargylamine (10 μL, 20 mg mL^−^^1^ in ethanol, final concentration 1.4 mM), phosphate buffer (332.5 µL, 100 mM potassium phosphate, pH 7.0, 200 mM NaCl), CuSO_4_ (2.5 µL, 20 mM in H_2_O), THPTA (tris(3-hydroxypropyltriazolylmethyl)amine, 5 µL, 50 mM in H_2_O) [39], aminoguanidine (25 µL, 100 mM in H_2_O), l-ascorbic acid (25 µL, 100 mM in H_2_O). The reaction mixture was incubated overnight at 4 °C and then extensively dialyzed.

## 4. Conclusions

The work described in this paper demonstrates the great potential of genetically engineering metabolic pathways to generate desired ncAAs inside the cell, and the possibility to bring it together with a reprogrammed protein translation apparatus. The combination of such metabolic engineering with *in vivo* incorporation of ncAAs has been indeed demonstrated few years ago by Mehl *et al.* [40]. In that work, the *Streptomyces venezuelae* gene-loci *pap*A, *pap*B and *pap*C, encoding the enzymes responsible for the production of *p*-aminophenylalanine as metabolic intermediate, were ‘borrowed’ and put into an *E. coli* strain that harboured a modified *M. jannaschii* tRNA_CUA_^Tyr^/TyrRS 21st pair specific for *p*-aminophenylalanine. Genes for these enzymes were imported by a low copy plasmid into *E. coli*, making the microbe capable of producing of *p*-aminophenylalanine from chorismate. The *p*-aminophenylalanine generated in the cell did not interfere with the bacterial metabolism and physiology, and was incorporated into proteins by the corresponding orthogonal pair. Similarly, Lepthien *et al*. [23] demonstrated the metabolic transformation of various indole analogs into their related tryptophan analogs in a Trp-auxotrophic *E. coli* by the endogenous action of tryptophan synthase; these analogs accumulated intracellularly [41] up to levels high enough for sufficient activation and charging onto tRNA_UGG_^Trp^ by the Trp-tRNA synthetase, and subsequently participated in ribosomal translation by recoding the UGG coding triplets.

Non-canonical amino acids that have been identified so far in natural products certainly represent only a small fraction of all chemically possible amino acids. This diversity can be further expanded to generate a pool of amino acids of anthropogenic origin, whose chemical and structural diversities transcend those found in nature. In this context, our work represents a novel approach for the synthesis and incorporation of ncAAs into recombinant proteins, combining metabolic engineering, bioorthogonal chemistry and ribosome-mediated integration of Met-analogs. In our methodology, l-azidohomoalanine is synthesized and incorporated into the selected target protein (barstar) within the bacterial host metabolism. Different forms of Aha-substituted recombinant barstar, either at *N*-terminal or internal positions were efficiently produced. All variants were purified, characterized by means of mass spectroscopy, fluorescence spectroscopy and circular dichroism, and showed the correct structural features. An *N*-terminal fluorescent derivative of Aha-barstar was synthesized by single step click chemistry cycloaddition of dansyl propargylamine, proving the system suitable for easy site-specific post-translational modifications of desired target proteins. Although Aha-ψB* derivatives had been already obtained by supplementation-based incorporation [33], in that previous case cells were directly fed with synthetically produced Aha. In our present effort, we improved the methodology through an artificial metabolism approach, to obligate *E. coli* cells to make Aha from an externally fed source of Oahs. A further likely step will be that to force *E. coli* cells to synthesize Aha and eventually other amino acid derivatives from common fermentable sources–such as glucose in minimal media–without the need to supply Oahs from the outside, by diverting the methionine biosynthetic pathway and drawing from central metabolites such as aspartate and TCA cycle intermediates.

## Abbreviations

ncAA: non-canonical amino acids
Aha: l-azidohomoalanine
Met: l-methionine
Oahs: *O*-acetyl-l-homoserine
PLP: pyridoxal 5'-phosphate
OAHSS: *O*-acetyl-l-homoserine sulfhydrylase
*cg*: *Corynebacterium glutamicum*
ψB*: ψ–bastar, “pseudo wild-type” form of barstar from *Bacillus amyloliquefaciens*
PMTs: posttranslational modifications

## Supplementary Materials

Supplementary materials can be accessed at: http://www.mdpi.com/1420-3049/19/1/1004/s1.
