# Supplementary material for: Coupling Bioorthogonal Chemistries with Artificial Metabolism: Intracellular Biosynthesis of Azidohomoalanine and Its Incorporation into Recombinant Proteins
Source: Molecules. 2014 Jan 15;19(1):1004–22. doi: 10.3390/molecules19011004 (PMC6271081; doi:10.3390/molecules19011004)

Supplementary Materials

Figure S1. NMR spectra for Oahs. (**a**) ^{1}H-NMR (D_2O, 400 MHz), (**b**) ^{13}C-NMR (D_2O, 100 MHz).

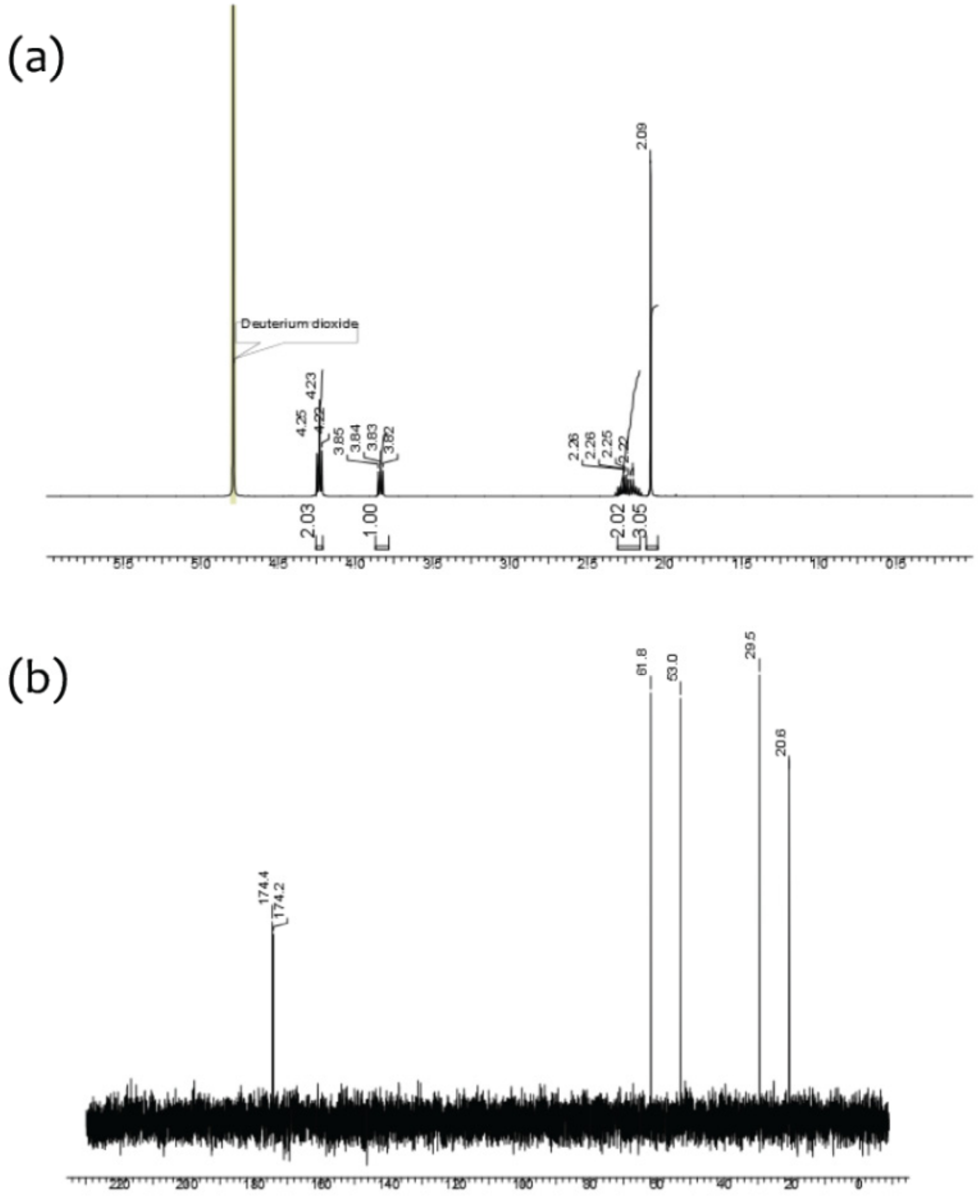

Figure S2. ESI-HRMS analysis of Oahs (positive mode).

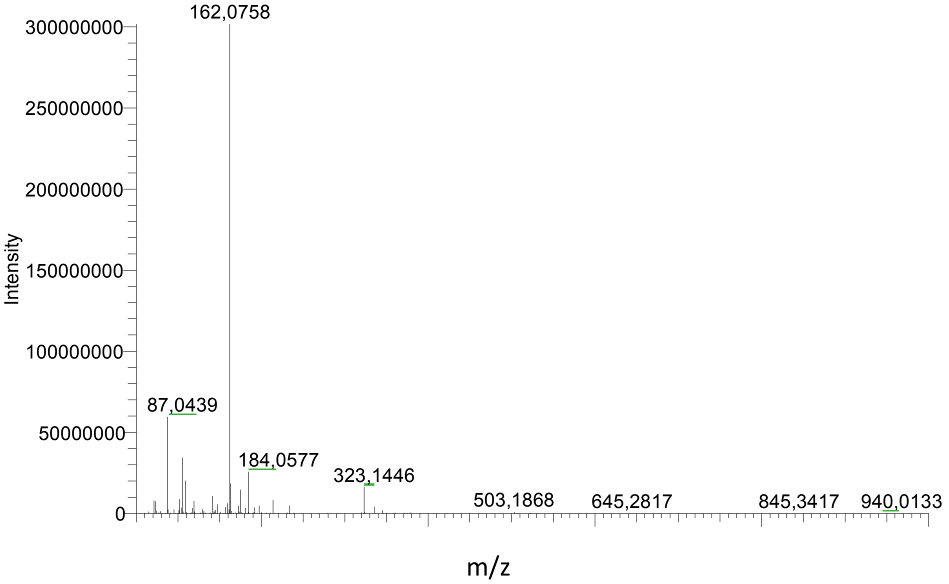

Figure S3. NMR spectra for Aha. (**a**) ^{1}H-NMR (D_2O, 400 MHz), (**b**) ^{13}C-NMR (D_2O, 100 MHz).

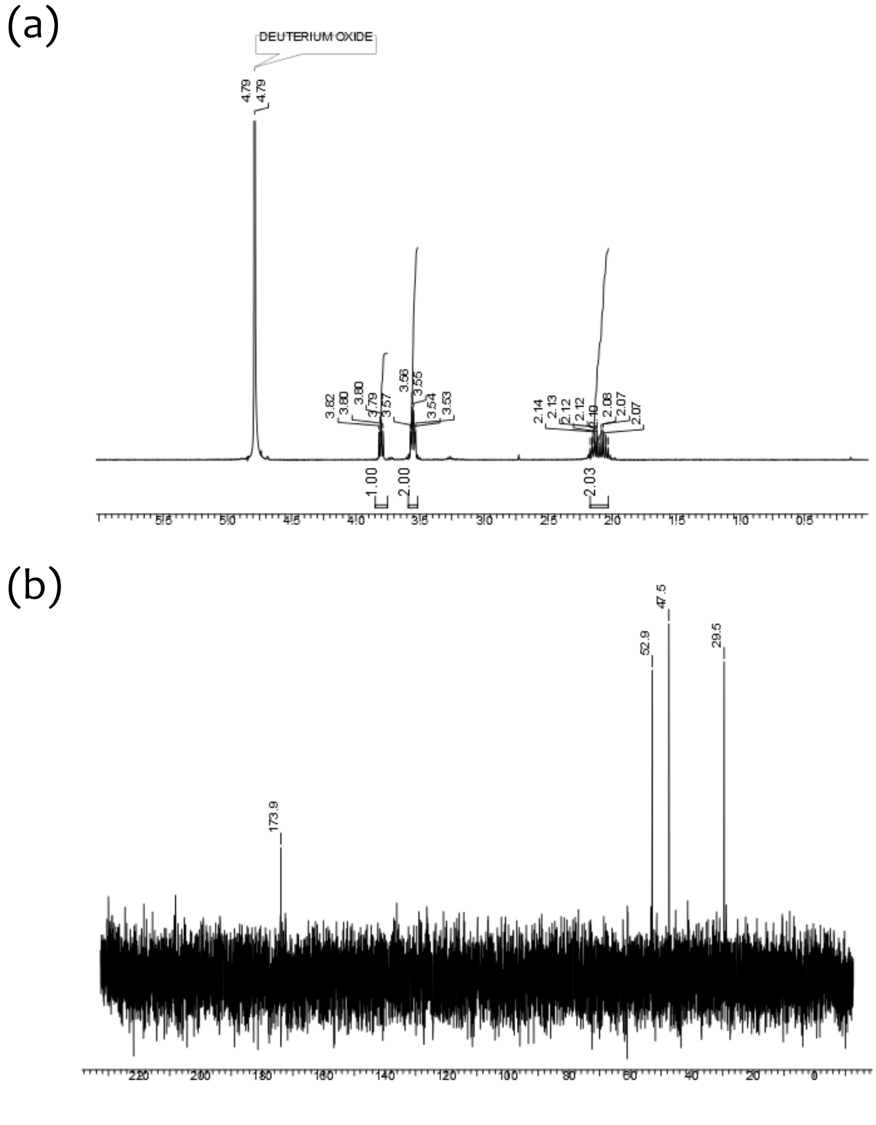

Figure S4. IR spectra of Oahs (**a**) and Aha (**b**). The black arrow in panel (**b**) indicates the strong 2110 cm^{-1} band typically attributed to azido (N_3) groups.

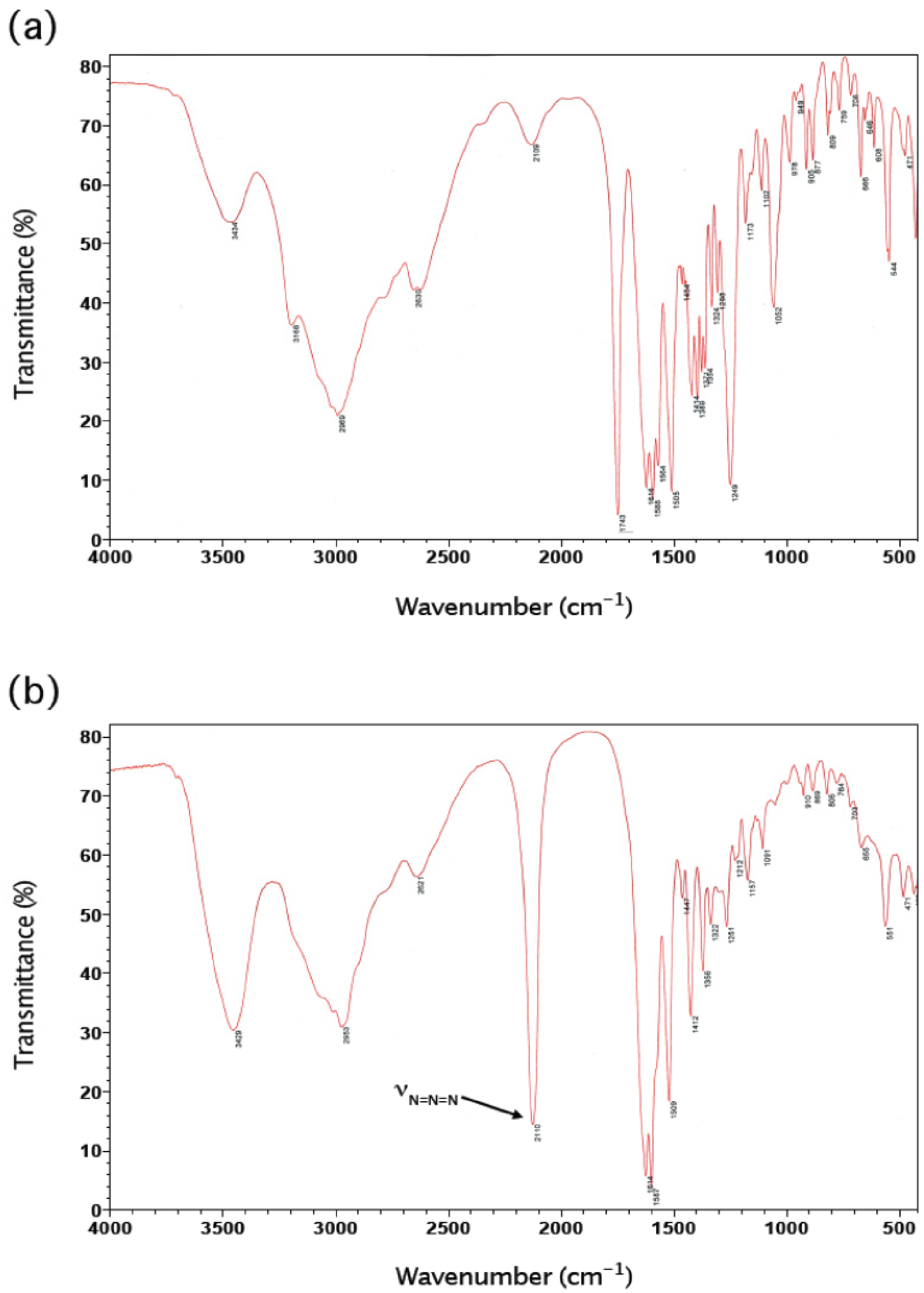

Supplement: Supplementary file 1 [file molecules-19-01004-s001.pdf]
